# A Novel and Cost-Effective Drive Circuit for Supplying a Piezoelectric Ceramic Actuator with Power-Factor-Correction and Soft-Switching Features

**DOI:** 10.3390/mi12101229

**Published:** 2021-10-09

**Authors:** Chun-An Cheng, Hung-Liang Cheng, Chien-Hsuan Chang, En-Chih Chang, Chih-Yang Tsai, Long-Fu Lan

**Affiliations:** Department of Electrical Engineering, I-Shou University, Kaohsiung City 84001, Taiwan; cacheng@isu.edu.tw (C.-A.C.); hlcheng@isu.edu.tw (H.-L.C.); chchang@isu.edu.tw (C.-H.C.); isu10701011m@cloud.isu.edu.tw (C.-Y.T.); isu11001004m@cloud.isu.edu.tw (L.-F.L.)

**Keywords:** drive circuit, power-factor-correction, zero-voltage switching, piezoelectric ceramic actuator

## Abstract

This paper proposes a novel and cost-effective drive circuit for supplying a piezoelectric ceramic actuator, which combines a dual boost AC-DC converter with a coupled inductor and a half-bridge resonant DC-AC inverter into a single-stage architecture with power-factor-correction (PFC) and soft-switching characteristics. The coupled inductor of the dual boost AC-DC converter sub-circuit is designed to work in discontinuous conduction mode (DCM), so the PFC function can be realized in the proposed drive circuit. The resonant tank of the half-bridge resonant inverter sub-circuit is designed as an inductive load, so that the two power switches in the presented drive circuit can achieve zero-voltage switching (ZVS) characteristics. A 50 W-rated prototype drive circuit providing a piezoelectric ceramic actuator has been successfully implemented in this paper. From the experimental results at 110 V input utility-line voltage, the drive circuit has the characteristics of high power factor and low input current total-harmonic-distortion factor, and two power switches have ZVS characteristics. Therefore, satisfactory outcomes from measured results prove the function of the proposed drive circuit.

## 1. Introduction

**Figure 1:**
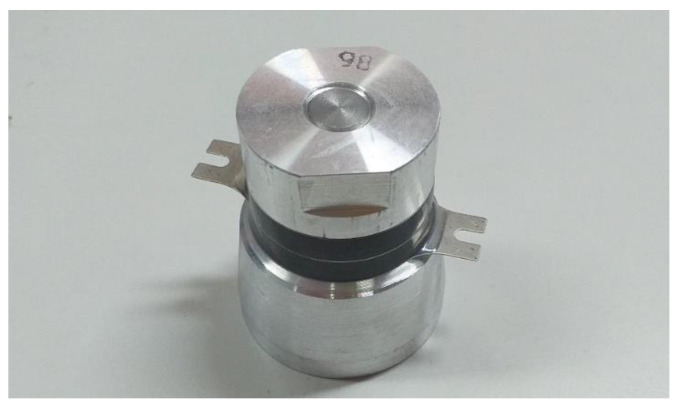
The photo of a piezoelectric ceramic actuator.

**Figure 2:**
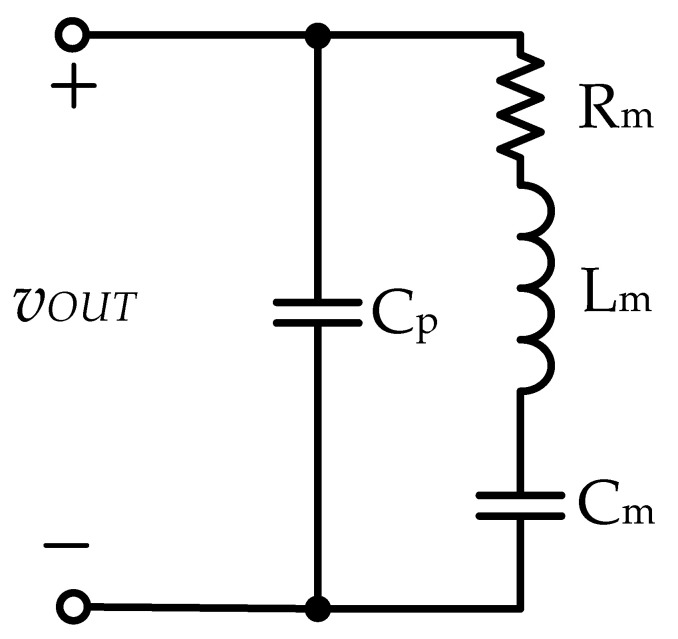
The equivalent circuit model for a piezoelectric ceramic actuator.

Piezoelectric ceramic actuators generate vibrations with a frequency above 20 kHz through the piezoelectric effect and have the characteristics of high accuracy, fast response, low power consumption, miniaturization, and high-density configuration. Piezoelectric ceramic actuators are widely used in low-power ultrasonic energy conversion circuits (such as ultrasonic beauty equipment, dental scalers, and atomizers) and high-power ultrasonic energy conversion circuits (such as ultrasonic cleaning machines, ultrasonic processing machines, and ultrasonic welding machines) [1,2,3,4,5,6,7]. Figure 1 shows a photo of a piezoelectric ceramic actuator. The equivalent circuit model of the piezoelectric ceramic actuator is shown in Figure 2, where the voltage source *v*_OUT_ is the voltage output from the driving circuit to the piezoelectric ceramic actuator; the capacitance *C*_p_ is the static capacitance of the piezoelectric ceramic actuator; the resistance *R*_m_ is the mechanical equivalent resistance; *L*_m_ is the mechanical equivalent inductance, and *C*_m_ is the mechanical equivalent capacitance [8,9,10,11].

**Figure 3:**
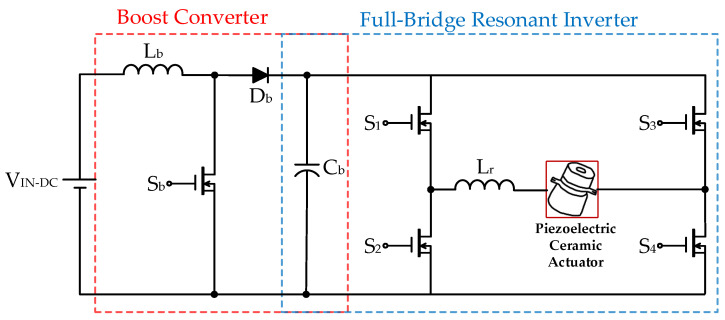
The conventional two-stage drive circuit for supplying a piezoelectric ceramic actuator applied with a DC input voltage source [12].

Figure 3 shows the conventional two-stage drive circuit for supplying a piezoelectric ceramic actuator applied with a DC input voltage source *V*_IN-DC_ [12], which consists of a front-stage DC-DC boost converter (including an inductor *L*_b_, a power switch *S*_b_, a diode *D*_b_ along with a DC-linked capacitor *C*_b_), and a rear-stage DC-AC full-bridge resonant converter (including four power switches *S*_1_, *S*_2_, *S*_3_, and *S*_4_ and a resonant inductor *L*_r_) that provides rated power to the piezoelectric ceramic actuator. 

**Figure 4:**
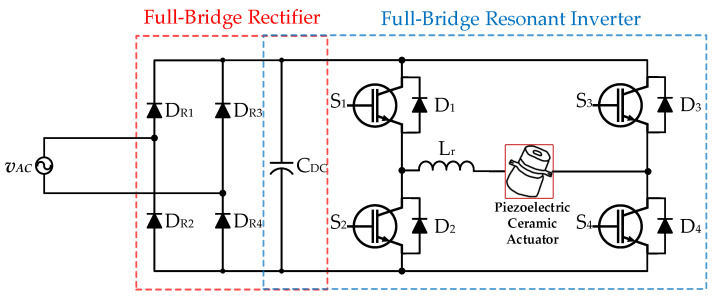
The conventional two-stage drive circuit for supplying a piezoelectric ceramic actuator applied with an AC input voltage source without PFC [13,14].

Figure 4 shows the conventional two-stage drive circuit for supplying a piezoelectric ceramic actuator applied with a AC input voltage source *v*_AC_ and without power-factor-correction (PFC) [13,14], which consists of a front-stage AC-DC full-bridge rectifier (including four diodes *D*_R1_, *D*_R2_, *D*_R3_, and *D*_R4_ along with a DC-linked capacitor *C*_DC_) and a rear-stage DC-AC full-bridge resonant converter (including four power switches *S*_1_, *S*_2_, *S*_3_, and *S*_4_; four diodes *D*_1_, *D*_2_, *D*_3_, and *D*_4_; and a resonant inductor *L*_r_) that provides rated power to the piezoelectric ceramic actuator. 

The traditional driving circuit that supplies power to the piezoelectric ceramic actuator requires more power switches, and the switching loss and conduction loss generated by the power switches are relatively large, which will affect the overall efficiency of the circuit. In response to these challenges, this paper presents a novel and cost-effective drive circuit for providing a piezoelectric ceramic actuator with PFC and soft-switching functions, which integrates a dual boost converter with a coupled inductor and a half-bridge resonant inverter. Descriptions of the operational modes and design equations along with experimental results of the proposed drive circuit are demonstrated in what follows.

## 2. The Proposed Drive Circuit for Supplying a Piezoelectric Ceramic Actuator

### 2.1. Introduction of Proposed Drive Circuit

**Figure 5:**
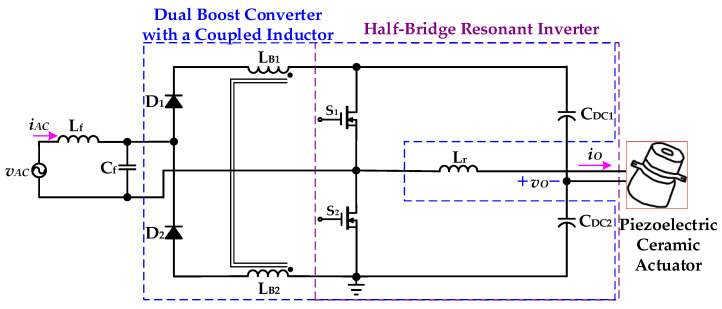
The proposed drive circuit for supplying a piezoelectric ceramic actuator with PFC.

Figure 5 shows the proposed drive circuit with PFC, which integrates a dual boost converter with a coupled inductor and a half-bridge resonant inverter, for supplying a piezoelectric ceramic actuator. The dual boost converter with a coupled-inductor sub-circuit, which is followed by an input AC voltage *v*_AC_ and a filter (*L*_f_ and *C*_f_), consists of two diodes (*D*_1_ and *D*_2_), a coupled-inductor (*L*_B1_ and *L*_B2_), two power switches (*S*_1_ and *S*_2_), and two DC-linked capacitors (*C*_DC1_ and *C*_DC2_). The half-bridge resonant inverter sub-circuit includes two switches (*S*_1_ and *S*_2_), two DC-linked capacitors (*C*_DC1_ and *C*_DC2_), and a resonant inductor (*L*_r_) along with the piezoelectric ceramic actuator. In addition, the coupled-inductor (*L*_B1_ and *L*_B2_) is designed to be operated in discontinuous-conduction mode (DCM) in order to accomplish input-current shaping. 

Table 1 shows comparisons between the conventional two-stage drive circuits in References [12,13,14] and the proposed single-stage one for the piezoelectric ceramic actuator. It can be seen that the proposed drive circuit has the characteristics of power-factor-correction and soft switching and requires fewer power switches than in References [12,13,14], so it can be a cost-effective alternative version for supplying a piezoelectric ceramic actuator.

### 2.2. Analysis of Operational Modes

When analyzing the operational modes of the piezoelectric ceramic actuator drive circuit, the assumptions made for some circuit components are as follows:

- The control signals of the power switches *S*_1_ and *S*_2_ are in a complementary state, and the essential diodes and parasitic capacitances on the power switches are considered.
- The two coupled inductors *L*_B1_ and *L*_B2_ in the drive circuit are designed to operate in discontinuous-conduction Mode (DCM).
- The equivalent resistance of diodes *D*_1_ and *D*_2_ and the forward bias voltage drop are ignored in the analysis.
- The remaining circuit components are assumed to be ideal.

**Figure 6:**
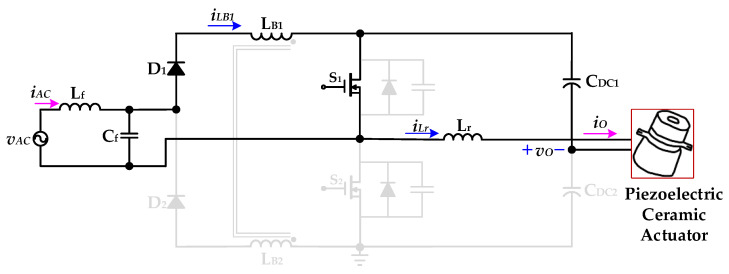
Operation *Mode 1* of the proposed drive circuit for the piezoelectric ceramic actuator.

**Figure 7:**
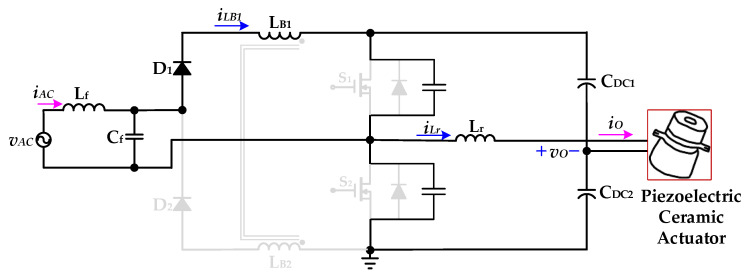
Operation *Mode 2* of the proposed drive circuit for the piezoelectric ceramic actuator.

**Figure 8:**
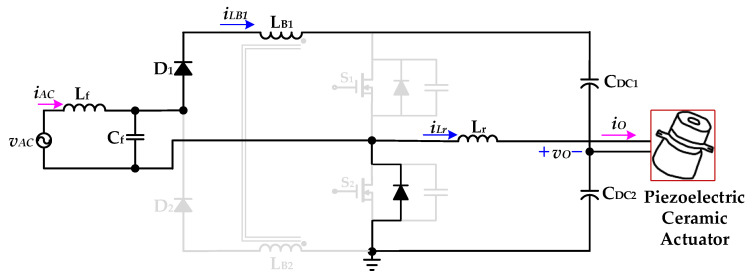
Operation *Mode 3* of the proposed drive circuit for the piezoelectric ceramic actuator.

**Figure 9:**
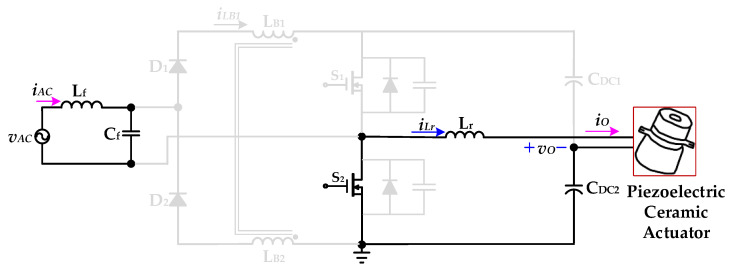
Operation *Mode 4* of the proposed drive circuit for the piezoelectric ceramic actuator.

**Figure 10:**
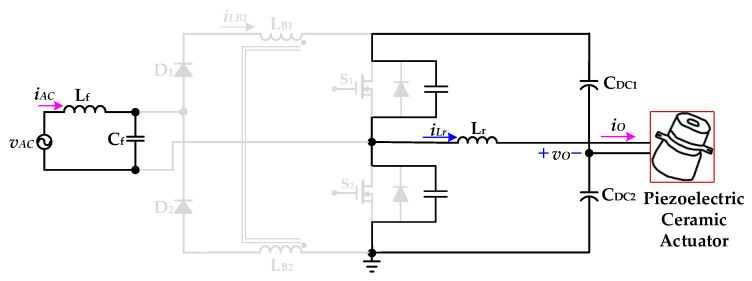
Operation *Mode 5* of the proposed drive circuit for the piezoelectric ceramic actuator.

**Figure 11:**
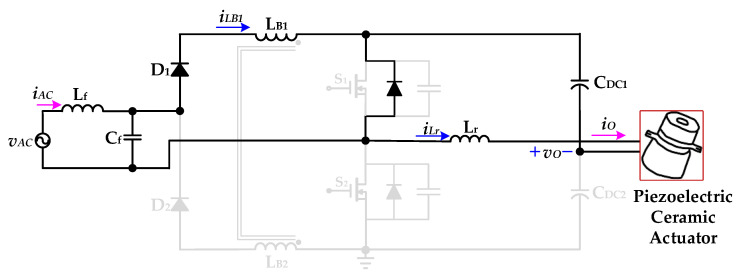
Operation *Mode 6* of the proposed drive circuit for the piezoelectric ceramic actuator.

**Figure 12:**
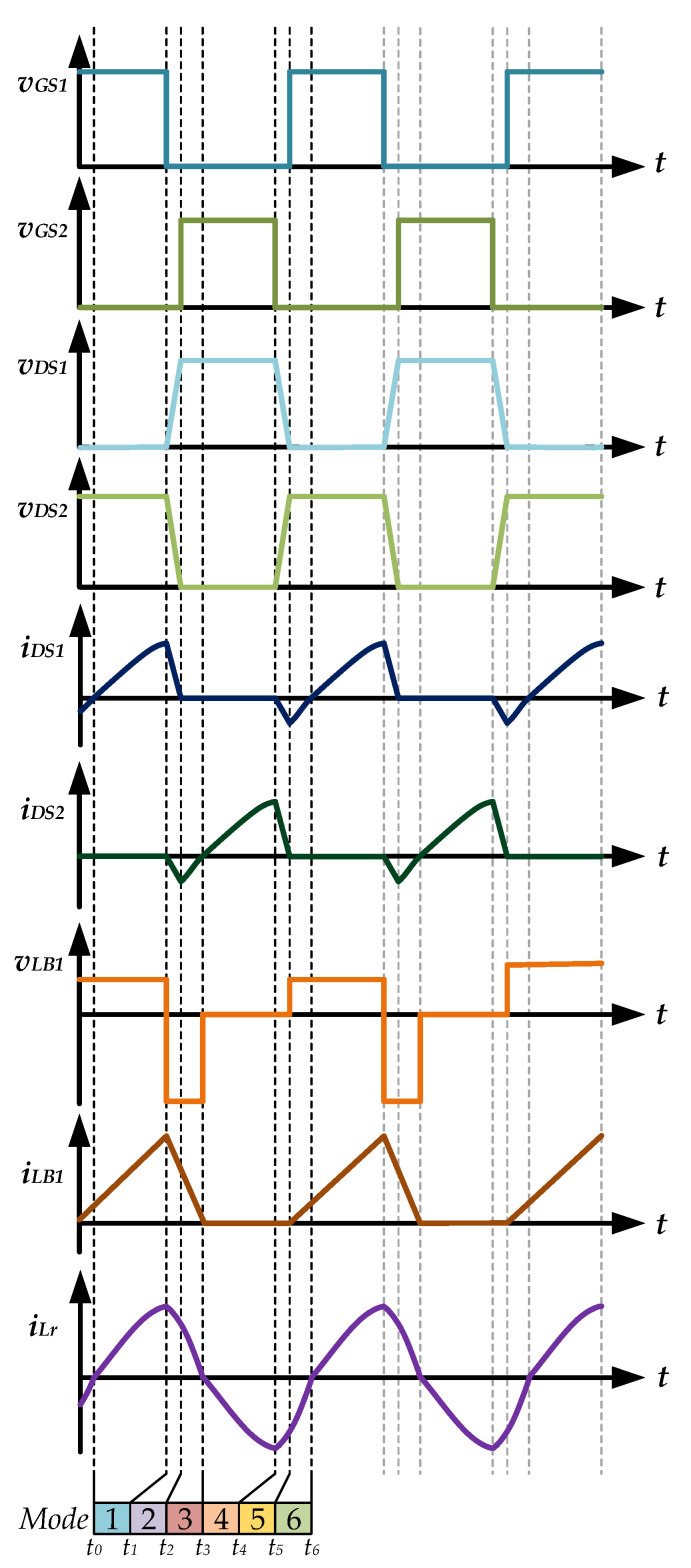
Theoretical waveforms of the proposed drive circuit of the piezoelectric ceramic actuator during the positive half-cycle of the utility-line voltage.

The operating modes and theoretical waveforms of the proposed piezoelectric ceramic driver drive circuit during the positive half-cycle of the utility-line voltage are shown in Figure 6, Figure 7, Figure 8, Figure 9, Figure 10, Figure 11 and Figure 12, respectively. The circuit analysis and operating modes of the drive circuit during the positive half-cycle of the utility-line voltage are described and discussed in detail below. 

#### 2.2.1. Operational *Mode 1* (t_0_ ≤ t < t_1_)

Figure 6 shows the operational *Mode 1* of the new piezoelectric ceramic actuator drive circuit with PFC. In the previous operation mode, the essential diode of the power switch *S*_1_ is forward-biased conduction. When the resonant inductor current *i*_Lr_ drops to zero, the power switch *S*_1_ is driven on and has ZVS. The voltage source *v*_AC_ provides energy to the coupled inductor *L*_B1_ through the inductance *L*_f_ and the capacitor *C*_f_ of the filter circuit, the diode *D*_1_ and the power switch *S*_1_, and the coupled inductor current *i*_LB1_ presents a linear increase. The DC-linked capacitor *C*_DC1_ charges the resonant inductor *L*_r_ through the power switch *S*_1_ and provides energy to the piezoelectric ceramic actuator. When the power switch *S*_1_ is turned off, the inductor current *i*_LB1_ rises to the maximum value. At time *t*_1_, *Mode 1* ends. 

#### 2.2.2. Operational *Mode 2* (t_1_ ≤ t < t_2_)

Figure 7 shows the operational *Mode 2* of the new piezoelectric ceramic actuator drive circuit with PFC. The voltage source *v*_AC_ provides energy to the parasitic capacitance of the power switch *S*_1_ through the inductance *L*_f_ and the capacitance *C*_f_ of the filter circuit, the diode *D*_1_, and the coupling inductor *L*_B1_, and the coupling inductor current *i*_LB1_ begins to decrease linearly. The DC-linked capacitor *C*_DC1_ and the resonant inductor *L*_r_ charge the parasitic capacitance of the power switch *S*_1_ and provide energy to the piezoelectric ceramic actuator. The parasitic capacitance of the power switch *S*_2_ and the resonant inductance *L*_r_ provide energy to the load and provide energy for the DC-linked capacitor *C*_DC2_. When the parasitic capacitance of the power switch *S*_2_ releases energy, the voltage *v*_DS2_ of the power switch *S*_2_ drops to zero, and the essential diode of the power switch *S*_2_ is forwardly biased and turned on. At time *t*_2_, *Mode 2* ends.

#### 2.2.3. Operational *Mode 3* (t_2_ ≤ t < t_3_) 

Figure 8 shows the operational *Mode 3* of the new piezoelectric ceramic actuator drive circuit with PFC. The voltage source *v*_AC_ and the coupled inductor *L*_B1_ charge the DC-linked capacitors *C*_DC1_ and *C*_DC2_ through the inductance *L*_f_ and capacitor *C*_f_ of the filter circuit, the diode *D*_1_, and the essential diode of the power switch *S*_2_. At this time, the coupled inductor current *i*_LB1_ shows a linear decrease. The resonant inductor *L*_r_ charges the DC-linked capacitor *C*_DC2_ through the essential diode of the power switch *S*_2_ and provides energy to the piezoelectric ceramic actuator. When the coupled inductor current *i*_LB1_ and the resonant inductor current *i*_Lr_ drop to zero, *Mode 3* ends.

#### 2.2.4. Operational *Mode 4* (t_3_ ≤ t < t_4_)

Figure 9 shows the operational *Mode 4* of the new piezoelectric ceramic actuator drive circuit with PFC. When the coupled inductor current *i*_LB1_ drops to zero, the power switch *S*_2_ is driven to turn on and has a ZVS characteristic. The DC-linked capacitor *C*_DC2_ charges the resonant inductor *L*_r_ through the power switch *S*_2_ and provides energy to the piezoelectric ceramic actuator. When the power switch *S*_2_ is turned off, *Mode 4* ends.

#### 2.2.5. Operational *Mode 5* (t_4_ ≤ t < t_5_)

Figure 10 shows the operational *Mode 5* of the new piezoelectric ceramic actuator drive circuit with PFC. The resonant inductor *L*_r_ and the parasitic capacitance of the power switch *S*_1_ charge the DC-linked capacitor *C*_DC1_ and provide energy to the piezoelectric ceramic actuator. At the same time, the resonant inductor *L*_r_ and the DC-linked capacitor *C*_DC2_ charge the parasitic capacitance of the power switch *S*_2_ and provide energy to the piezoelectric ceramic actuator. When the parasitic capacitance energy of the power switch *S*_1_ is released and the voltage *v*_DS1_ of the power switch *S*_1_ drops to zero, the essential diode of the power switch *S*_1_ is forwardly biased and turned on. At time *t*_5_, *Mode 5* completes.

#### 2.2.6. Operational *Mode 6* (t_5_ ≤ t < t_6_)

Figure 11 shows the operational *Mode 6* of the new piezoelectric ceramic actuator drive circuit with PFC. In the previous operational mode, the energy of the parasitic capacitance of the power switch *S*_1_ is released, the voltage *v*_DS1_ of the power switch *S*_1_ drops to zero, and the essential diode of the power switch *S*_1_ is turned on in a forward bias. The voltage source *v*_AC_ provides energy to the coupled inductor *L*_B1_ through the inductance *L*_f_ and the capacitor *C*_f_ of the filter circuit and the diode *D*_1_, and the coupled inductor current *i*_LB1_ rises linearly from zero. In addition, through the essential diode of the power switch *S*_1_, the resonant inductor *L*_r_ and the voltage source *v*_AC_ provide energy to the DC-linked capacitor *C*_DC1_ and the piezoelectric ceramic actuator. When the resonant inductor current *i*_Lr_ drops to zero and the power switch *S*_1_ is driven and turned on, *Mode 6* ends and the circuit operation returns to *Mode 1*.

In addition, Table 2 shows states of the main power devices in each operational mode during the positive half-cycle of the utility-line voltage.

### 2.3. Design Equations of Key Circuit Parameters

#### 2.3.1. Design Equation of the Coupled Inductors *L*_B1_ and *L*_B2_

**Figure 13:**
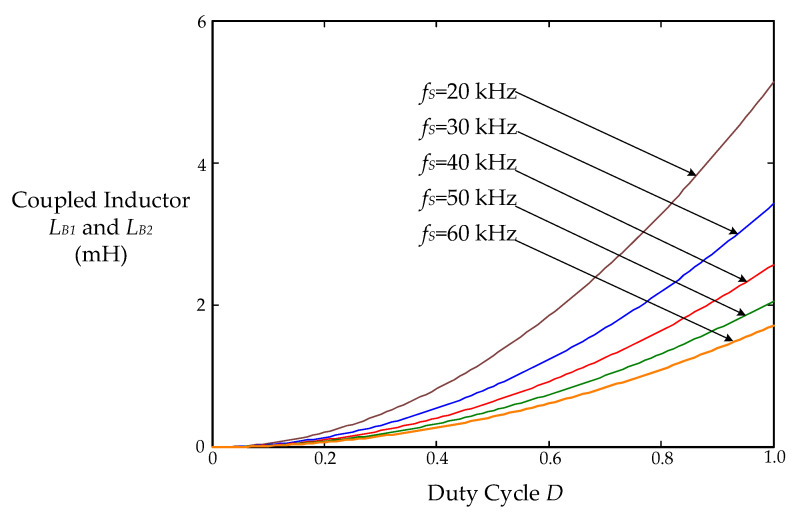
The relationship between the coupled inductors *L*_B1_ and *L*_B2_ and the duty cycle *D* at different switching frequencies *f*_S_.

The design equation of the coupled inductors *L*_B1_ and *L*_B2_ can be represented by [15]:

(1) $$L_{B1}=\frac{\eta v_{\mathrm{AC}\text{-}\mathrm{rms}}{}^{2}D^{2}}{2P_{O}f_{S}}=L_{B2}$$

where *η* is the estimated efficiency of the proposed drive circuit; *v*_AC-rms_ is the root-mean-square (rms) value of the input utility-line voltage *v*_AC_; *D* and *f*_S_ are the duty ratio and switching frequency of the power switches, respectively; *P*_O_ is the output power. From the Formula (1), it can be drawn that Figure 13 shows the relationship between the coupled inductors *L*_B1_ and *L*_B2_ and the duty cycle *D* at different switching frequencies *f*_S_.

With a *η* of 0.8, a *v*_AC-rms_ of 110 V, a *D* of 0.5, a *P*_O_ of 50 W, and a switching frequency *f*_S_ of 40 kHz, the inductances of the coupled inductors *L*_B1_ and *L*_B2_ are calculated as

$$L_{B1}=L_{B2}=\frac{\eta v_{\mathrm{AC}\text{-}\mathrm{rms}}{}^{2}D^{2}}{2P_{O}f_{S}}=\frac{0.8\cdot 110^{2}\cdot 0.5^{2}}{2\cdot 50\cdot 40k}=650\;\mu H$$

In addition, the coupled inductors *L*_B1_ and *L*_B2_ in the prototype drive circuit are 500 μH.

#### 2.3.2. Design Equation of the Resonant Inductor *L*_r_

**Figure 14:**
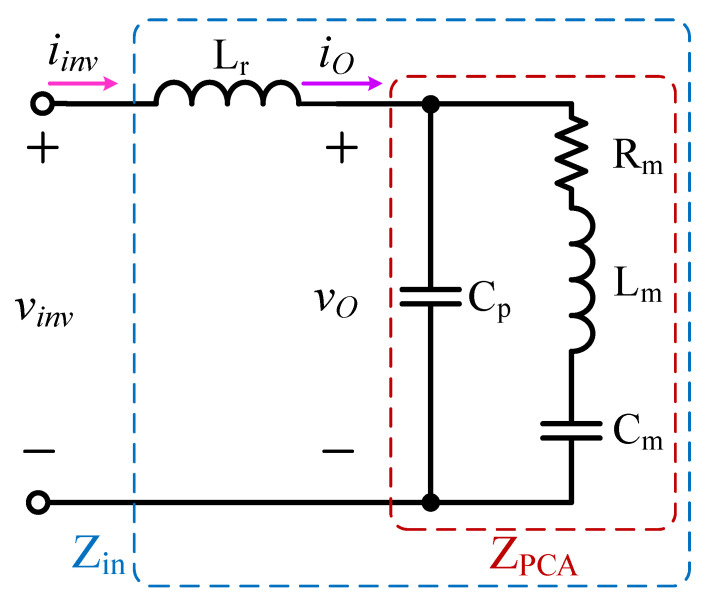
The equivalent circuit diagram of the resonant tank circuit combined with the piezoelectric ceramic actuator circuit model.

Figure 14 shows the equivalent circuit diagram of the resonant inductor combined with the piezoelectric ceramic actuator circuit model; *v*_inv_ and *i*_inv_ respectively represent the input voltage and current of the equivalent circuit; *Z*_PCA_ represents the equivalent circuit model of the piezoelectric ceramic actuator; *Z*_in_ represents the input impedance of the equivalent circuit. The output power *P*_O_ of the piezoelectric ceramic actuator is provided by the fundamental component of the input current *i*_inv_ of the resonant tank circuit, and the switching frequency *f*_S_ of the power switch is designed to be equal to the resonant frequency *f*_r_ of the piezoelectric ceramic actuator. In addition, at the resonance frequency of the piezoelectric ceramic actuator, the equivalent series impedance in the right branch of the *Z*_PCT_ resonance tank circuit is reduced to only the resistance *R*_m_. The rms value *I*_inv1-rms_ of the fundamental component of the current *i*_inv_ can be expressed as [13]

(2) $$I_{\mathrm{inv}1\text{-}\mathrm{rms}}=\frac{R_{m}-j\left(\frac{1}{2\pi f_{r}C_{p}}\right)}{-j\left(\frac{1}{2\pi f_{r}C_{p}}\right)}\left(\sqrt{\frac{P_{O}}{R_{m}}}\right)$$

The input impedance *Z*_in_ of the equivalent circuit is expressed as

(3) $$Z_{\mathrm{in}}=Z_{\mathrm{PCA}}+jZ_{\mathrm{Lr}}=(R_{1}+jX_{1})+j2\pi f_{r}L_{r}$$

where *R*_1_ and *X*_1_ are the equivalent resistance and reactance of the piezoelectric ceramic actuator impedance *Z*_PCA_, and they can be respectively expressed as [13]

(4) $$R_{1}=\frac{R_{m}}{1+{\left(2\pi f_{r}\right)}^{2}C_{p}^{2}R_{m}^{2}}$$

(5) $$X_{1}=\frac{2\pi f_{r}C_{p}R_{m}^{2}}{1+{\left(2\pi f_{r}\right)}^{2}C_{p}^{2}R_{m}^{2}}$$

After dividing the maximum value of the input voltage *V*_inv1-max_ by the maximum value of the input current *√2I*_inv1-rms_ of the equivalent circuit, the amplitue of the input impedance *Z*_in_ can be expressed as

(6) $${|Z}_{in}|=\frac{V_{\mathrm{inv}1\text{-}\max}}{\sqrt{2}I_{\mathrm{invl}\text{-}\mathrm{rms}}}=\frac{{8V}_{\mathrm{DC}}}{\sqrt{2}\pi I_{\mathrm{invl}\text{-}\mathrm{rms}}}$$

where *V*_inv1-max_ is the maximum level of the fundamental component *V*_inv1_ of the input voltage *v*_inv_ of the resonant tank circuit; *V*_DC_ is the voltage level of the DC-linked capacitors *C*_DC1_ and *C*_DC2_.

By combining (3) with (6), the design formula of the resonant inductor *L*_r_ can be expressed as [13]

(7) $$L_{r}=\frac{1}{2\pi f_{r}}\left(X_{1}+\sqrt{|Z_{\mathrm{in}}{|}^{2}-R_{1}^{2}}\right)$$

With a *R*_m_ of 25 Ω, a *C*_p_ of 4000 pF, a *P*_O_ of 50 W, and a resonant frequency *f*_r_ of 40 kHz, the parameter *I*_inv1-rms_ is calculated as

$$I_{\mathrm{inv}1\text{-}\mathrm{rms}}=\frac{R_{m}-j\left(\frac{1}{2\pi f_{r}C_{p}}\right)}{-j\left(\frac{1}{2\pi f_{r}C_{p}}\right)}\left(\sqrt{\frac{P_{O}}{R_{m}}}\right)=\frac{25-j\left(\frac{1}{2\pi \cdot 40k\cdot 4000p}\right)}{-j\left(\frac{1}{2\pi \cdot 40k\cdot 4000p}\right)}\left(\sqrt{\frac{50}{25}}\right)=1.414∠1.432^{0}A$$

The parameters *R*_1_ and *X*_1_ are respectively calculated as

$$R_{1}=\frac{R_{m}}{1+{\left(2\pi f_{r}\right)}^{2}C_{p}^{2}R_{m}^{2}}=\frac{25}{1+{\left(2\pi \cdot 40k\right)}^{2}\cdot {\left(4000p\right)}^{2}\cdot 25^{2}}=24.98\;\Omega$$

$$X_{1}=\frac{2\pi f_{r}C_{p}R_{m}^{2}}{1+{\left(2\pi f_{r}\right)}^{2}C_{p}^{2}R_{m}^{2}}=\frac{2\pi \cdot 40k\cdot 4000p\cdot 25^{2}}{1+{\left(2\pi \cdot 40k\right)}^{2}\cdot {\left(4000p\right)}^{2}\cdot 25^{2}}=0.625\;\Omega$$

With a *V*_DC_ of 700 V and a *I*_rnv1-rms_ of 1.414 A, the parameter |*Z*_in_*|* is calculated as

$${|Z}_{in}|=\frac{V_{\mathrm{inv}\text{-}\max}}{\sqrt{2}I_{\mathrm{invl}\text{-}\mathrm{rms}}}=\frac{{8V}_{\mathrm{DC}}}{\sqrt{2}\pi I_{\mathrm{invl}\text{-}\mathrm{rms}}}=\frac{8\cdot 700}{\sqrt{2}\pi \cdot 1.414}=891.4\;\Omega$$

Therefore, the parameter *L*_r_ is calculated as

$$L_{r}=\frac{1}{2\pi f_{r}}\left(X_{1}+\sqrt{|Z_{\mathrm{in}}{|}^{2}-R_{1}^{2}}\right)=\frac{1}{2\pi \cdot 40k}\left(0.625+\sqrt{891.4^{2}-24.98^{2}}\right)=3.55\;\mathrm{mH}$$

In addition, the resonance inductor *L*_r_ in the prototype drive circuit is 3.95 mH.

#### 2.3.3. Design of Input Low-Pass Filter

A low-pass filter is usually added to the AC input power terminal, which is composed of an inductor *L*_f_ and a capacitor *C*_f_. The cut-off frequency *f*_cut-off_ of the input low-pass filter is represented by

(8) $$f_{\mathrm{cut}\text{-}\mathrm{off}}=\frac{1}{2\pi \sqrt{L_{f}C_{f}}}$$

In order to filter high-frequency switching noise, the design consideration of the cut-off frequency *f*_cut-off_ of the input low-pass filter is determined as one-tenth of the switching frequency *f*_S_. Rearranging (8), the design equation of the inductor *L*_f_ is given by

(9) $$L_{f}=\frac{1}{4\pi^{2}f_{\mathrm{cut}\text{-}\mathrm{off}}{}^{2}C_{f}}$$

With a cut-off frequency *f*_cut-off_ of 4 kHz and selecting a capacitor *C*_f_ of 470 nF, the inductor *L*_f_ is determined by

$$L_{f}=\frac{1}{4\pi^{2}f_{\mathrm{cut}\text{-}\mathrm{off}}{}^{2}C_{f}}=\frac{1}{4\pi^{2}\cdot {\left(4\mathrm{kHz}\right)}^{2}\cdot 470n}=3.36\;\mathrm{mH}$$

## 3. Experimental Results of the Proposed Drive Circuit

**Figure 15:**
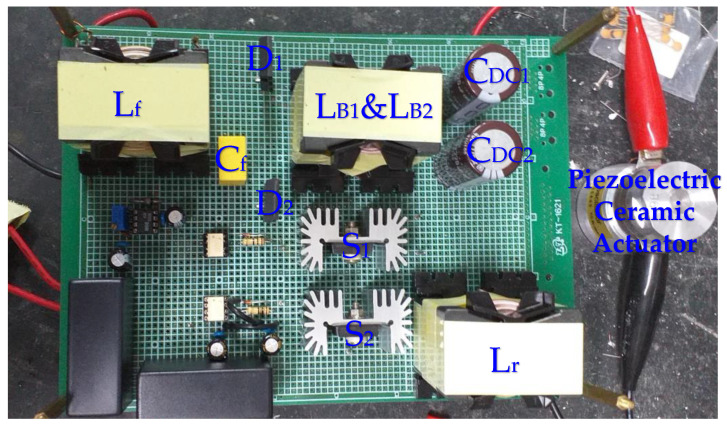
A photograph of the proposed prototype drive circuit for supplying a piezoelectric ceramic actuator.

In this paper, a prototype of the proposed drive circuit for supplying a 50 W-rated piezoelectric ceramic actuator has already been implemented and testified. A photograph of the proposed prototype drive circuit for supplying a piezoelectric ceramic actuator is shown in Figure 15, and the key circuit components have been indicated in the photograph. The parameters of the utilized piezoelectric ceramic actuator are shown in Table 3. In addition, the components utilized in the prototype drive circuit for the piezoelectric ceramic actuator are shown in Table 4. 

**Figure 16:**
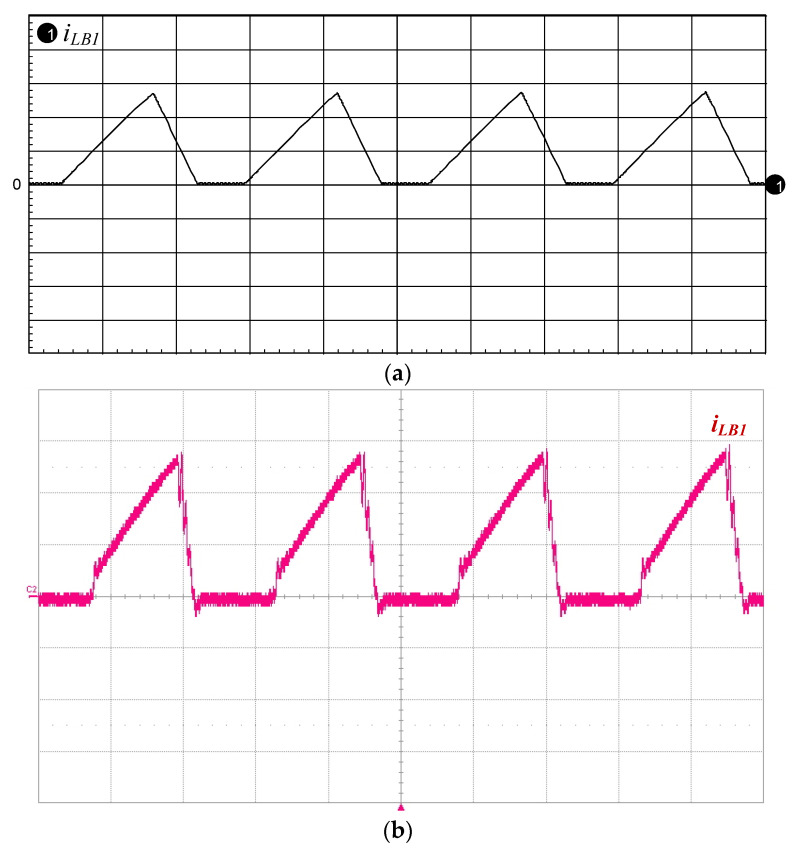
(**a**) Simulated and (**b**) measured inductor current *i*_LB1_ (1 A/div); time scale: 10 μs/div.

**Figure 17:**
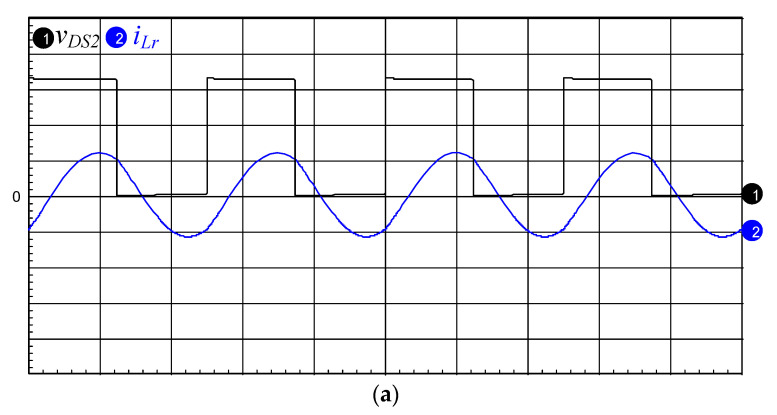

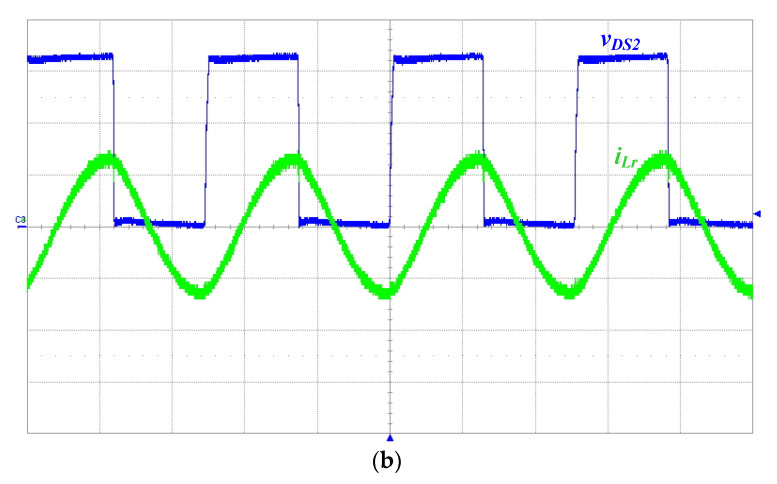
(**a**) Simulated and (**b**) measured switch voltage *v*_DS2_ (200 V/div) and resonant inductor current *i*_Lr_ (1 A/div); time scale: 10 μs/div.

**Figure 18:**
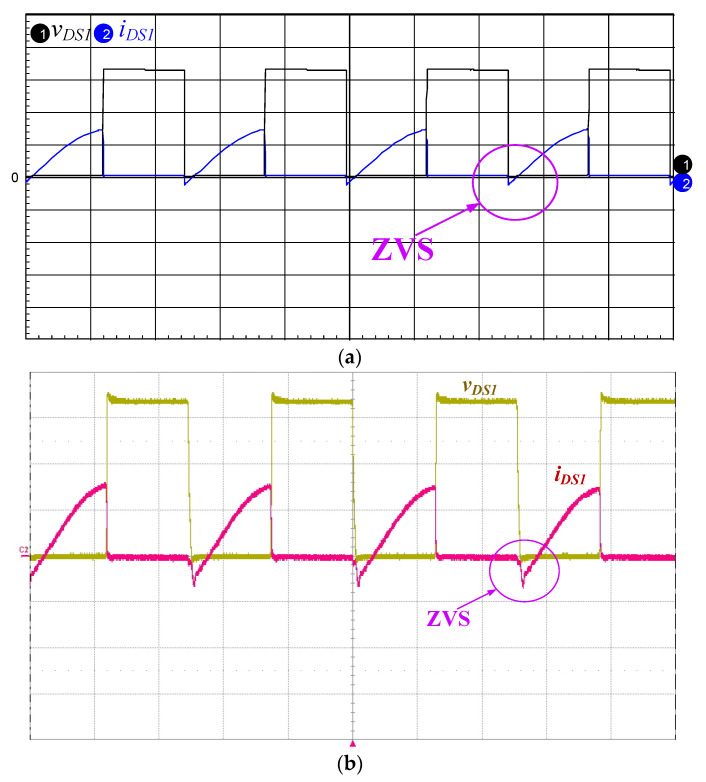
(**a**) Simulated and (**b**) measured switch voltage *v*_DS1_ (200 V/div) and switch current *i*_DS1_ (2 A/div); time scale: 10 μs/div.

**Figure 19:**
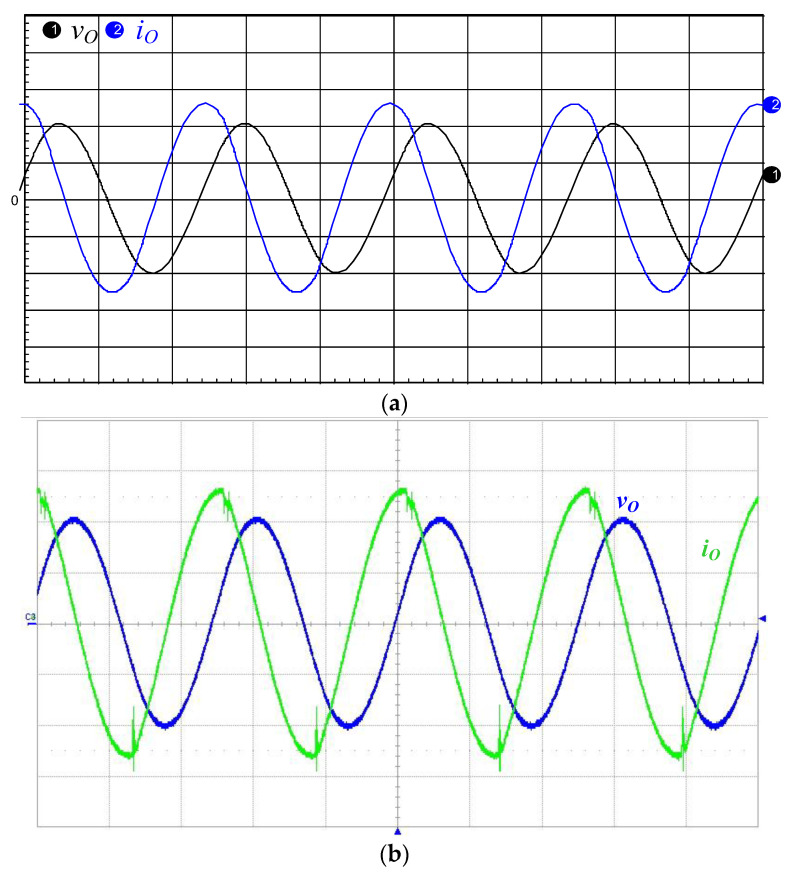
(**a**) Simulated and (**b**) measured output voltage *v*_O_ (500 V/div) and current *i*_O_ (0.5 A/div); time scale: 10 μs/div.

Figure 16a,b presents the simulated and measured inductor current *i*_LB1_, and it can be seen that the current *i*_LB1_ is operated in DCM. Figure 17a,b shows the simulated and measured switch voltage *v*_DS2_ and resonant inductor current *i*_Lr_. It can be seen that the inductor current *i*_Lr_ lags with respect to voltage *v*_DS2_ so that the series resonant circuit is similar to an inductive load. Figure 18a,b presents the simulated and measured switch voltage *v*_DS1_ and switch current *i*_DS1_; thus, ZVS occurred on the power switch for lowering the switching losses. Figure 19a,b depicts the simulated and measured output voltage *v*_O_ and output current *i*_O_. It can be seen from the waveform that the output voltage *v*_O_ lags the output current *i*_O_, so the piezoelectric ceramic actuator has capacitive characteristics.

**Figure 20:**
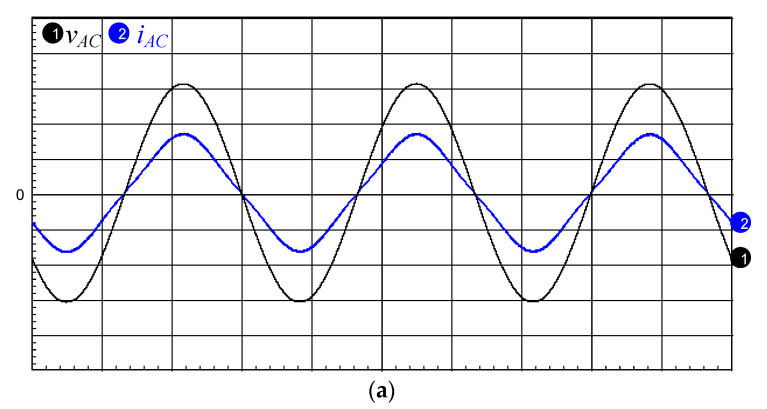

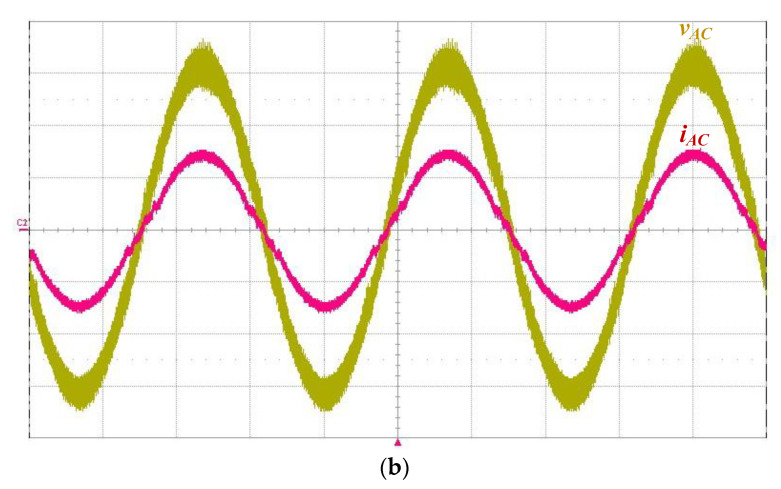
(**a**) Simulated and (**b**) measured input utility-line voltage *v*_AC_ (50 V/div) and current *i*_AC_ (1 A/div); time scale: 5 ms/div.

**Figure 21:**
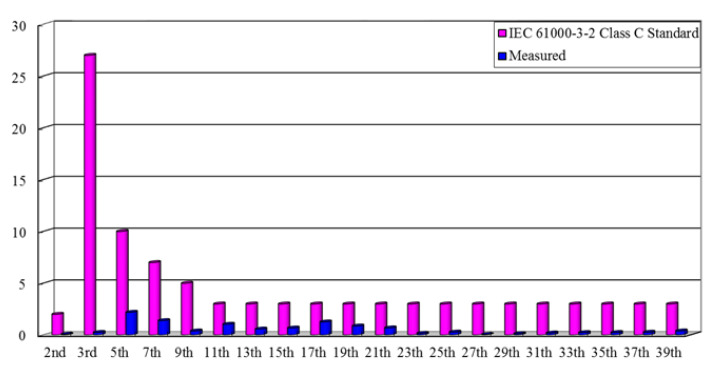
Measured harmonics of the input utility-line current in comparison with the IEC 61000-3-2 class C standard.

The simulated and measured waveforms of input utility-line voltage *v*_AC_ and current *i*_AC_ are respectively shown in Figure 20a,b, and it can be seen that PFC is achieved in the proposed drive circuit. Figure 21 shows the use of a power analyzer (Tektronix PA 4000) to measure the harmonic components of the AC input current and compare it with the IEC 61000-3-2 class C standard. From the figure, it is known that all current harmonics meet the requirements. Additionally, the measured power factor and the input utility-line current total-harmonic distortion (THD) of the proposed drive circuit are 0.8683 and 3.4927%, respectively.

## 4. Conclusions

This paper proposes a novel and cost-effective drive circuit, which combines a dual boost converter with a coupled inductor and a half-bridge resonant inverter, with PFC and soft-switching features for providing a piezoelectric ceramic actuator. A 50 W-rated prototype drive circuit has been implemented and tested with an input utility-line voltage of 110 V. From the experimental results at a 110 V input utility-line voltage, the driving circuit developed in this thesis has as characteristics a high power factor (>0.86) and a low input current total-harmonic-distortion factor (<4%), and two power switches possess the ZVS feature.

## Figures and Tables

**Table 1:** Comparisons between the conventional drive circuits and the proposed version for a piezoelectric ceramic actuator.

Item	Conventional Two-Stage Drive Circuit [12]	Conventional Two-Stage Drive Circuit [13,14]	Proposed Single-Stage Drive Circuit
Number of Required Power Switches	5	4	2
Number of Required Diodes	1	8	2
Number of Required Capacitors	1	1	3
Number of Required Magnetic Components	2	1	3
Input Voltage Source Suitable for the Application	DC Voltage	AC Voltage	AC Voltage
Function of Power-Factor-Correction	Not Available	No	Yes
Soft-Switching of Power Switches	Not All Switches	Yes (All Switches)	Yes (All Switches)

**Table 2:** States of the main power devices in each operational mode during the positive half-cycle of the utility-line voltage.

Main Power Devices	*Mode 1*	*Mode 2*	*Mode 3*	*Mode 4*	*Mode 5*	*Mode 6*
Switch *S*_1_	On	Off	Off	Off	Off	Off
Switch *S*_2_	Off	Off	Off	On	Off	Off
Diode *D*_1_	On	On	On	Off	Off	On
Diode *D*_2_	Off	Off	Off	Off	Off	Off
Inductor *L*_B1_	Charging	Discharging	Discharging	Discharging	Not Available	Charging
Inductor *L*_B2_	Not Available	Not Available	Not Available	Not Available	Not Available	Not Available
Inductor *L*_r_	Charging	Discharging	Discharging	Discharging	Discharging	Charging
Capacitor *C*_DC1_	Discharging	Discharging	Charging	Not Available	Discharging	Charging
Capacitor *C*_DC2_	Not Available	Charging	Charging	Discharging	Discharging	Not Available

**Table 3:** Parameters of the utilized piezoelectric ceramic actuator.

Parameter	Value
Resonant Frequency *f*_r_	40 kHz
Mechanical Equivalent Resistance *R*_m_	25 Ω
Static Capacitance *C*_p_	4000 pF
Rated Power *P*_O_	50 W

**Table 4:** Components utilized in the prototype of the proposed drive circuit.

Parameter/Component	Value
Diode *D*_1_, *D*_2_	MUR460
Filter Inductor *L*_f_	3.36 mH
Filter Capacitor *C*_f_	470 nF
Coupled Inductor *L*_B1_, *L*_B2_	500 μH
DC-linked Capacitor *C*_DC1_, *C*_DC2_	220 μF
Power Switches *S*_1_, *S*_2_	W12NK90Z
Resonant Inductor *L*_r_	3.95 mH
